# Supplementary material for: A multicenter analytical performance evaluation of a multiplexed immunoarray for the simultaneous measurement of biomarkers of micronutrient deficiency, inflammation and malarial antigenemia
Source: PLoS One. 2021 Nov 4;16(11):e0259509. doi: 10.1371/journal.pone.0259509 (PMC8568126; doi:10.1371/journal.pone.0259509)
Supplement: S1 Table — Within and between tube variation in measures from 19 aliquots for each of two samples selected at random from aliquots prepared for distribution to partner labs. CVs were calculated using the Rodbard variance components model. CV for HRP2 was not calculated because results were above the upper limit of detection. CV, coefficient of variation; AGP, α-1-acid glycoprotein; CRP, C-reactive protein; HRP2, histidine rich protein 2; N/A, not available; RBP4, retinol binding protein 4; sTfR, soluble transferrin receptor; Tg, thyroglobulin. (DOCX) [file pone.0259509.s001.docx]

**S1 Table: Assay precision for two specimens across 19 replicate aliquots.**

| **Analyte** | **Sample number** | **Mean concentration** | **%CV within tube** | **%CV between tubes** |
| --- | --- | --- | --- | --- |
| AGP (g/L) | 1 | 0.68 | 1.84 | 3.23 |
|  | 2 | 0.63 | 2.53 | 2.48 |
| CRP (mg/L) | 1 | 19.34 | 5.53 | 2.01 |
|  | 2 | 6.39 | 6.52 | 4.60 |
| Ferritin (µg/L) | 1 | 170.00 | 4.85 | 6.41 |
|  | 2 | 107.16 | 5.76 | 4.07 |
| HRP2 (µg/L) | 1 | >8.0 | N/A | N/A |
|  | 2 | >8.0 | N/A | N/A |
| RBP4 (µmol/L) | 1 | 1.23 | 6.90 | 6.65 |
|  | 2 | 1.32 | 3.16 | 1.71 |
| sTfR (mg/L) | 1 | 19.83 | 3.60 | 5.22 |
|  | 2 | 15.49 | 3.77 | 2.67 |
| Tg (µg/L) | 1 | 14.67 | 8.68 | 8.30 |
|  | 2 | 28.28 | 3.67 | 2.60 |

Within and between tube variation in measures from 19 aliquots for each of two samples selected at random from aliquots prepared for distribution to partner labs. CVs were calculated using the Rodbard variance components model. CV for HRP2 was not calculated because results were above the upper limit of detection. CV, coefficient of variation; AGP, α-1-acid glycoprotein; CRP, C-reactive protein; HRP2, histidine rich protein 2; N/A, not available; RBP4, retinol binding protein 4; sTfR, soluble transferrin receptor; Tg, thyroglobulin.
