# Supplementary material for: A multicenter analytical performance evaluation of a multiplexed immunoarray for the simultaneous measurement of biomarkers of micronutrient deficiency, inflammation and malarial antigenemia
Source: PLoS One. 2021 Nov 4;16(11):e0259509. doi: 10.1371/journal.pone.0259509 (PMC8568126; doi:10.1371/journal.pone.0259509)
Supplement: S2 Table — The expected value for each biomarker is shown in addition to the acceptable values for upper and lower limits. AGP, α-1-acid glycoprotein; CRP, C-reactive protein; HRP2, histidine rich protein 2; N/A, not available; RBP4, retinol binding protein 4; sTfR, soluble transferrin receptor; Tg, thyroglobulin. (DOCX) [file pone.0259509.s002.docx]

**S2 Table. The established values for the G and H controls developed for the 7-plex array.**

| Analyte | Control | Expected Value | Lower Limit | Upper Limit |
| --- | --- | --- | --- | --- |
| AGP (g/L) | G | 0.10 | 0.07 | 0.13 |
|  | H | 0.02 | 0.015 | 0.03 |
| CRP (mg/L) | G | 0.87 | 0.67 | 1.07 |
|  | H | 0.07 | 0.05 | 0.09 |
| Ferritin (µg/L) | G | 22.1 | 15.5 | 28.8 |
|  | H | 1.21 | 0.82 | 1.61 |
| HRP2 (µg/L) | G | N/A | None | None |
|  | H | 0.016 | 0.009 | 0.023 |
| RBP4 (µmol/L) | G | 0.26 | 0.18 | 0.33 |
|  | H | 0.04 | 0.03 | 0.05 |
| sTfR (mg/L) | G | 0.78 | 0.41 | 1.15 |
|  | H | 0.23 | 0.16 | 0.29 |
| Tg (µg/L) | G | 0.48 | 0.31 | 0.65 |
|  | H | 0.06 | 0.02 | 0.10 |

The expected value for each biomarker is shown in addition to the acceptable values for upper and lower limits. AGP, α-1-acid glycoprotein; CRP, C-reactive protein; HRP2, histidine rich protein 2; N/A, not available; RBP4, retinol binding protein 4; sTfR, soluble transferrin receptor; Tg, thyroglobulin.
