# Supplementary material for: A multicenter analytical performance evaluation of a multiplexed immunoarray for the simultaneous measurement of biomarkers of micronutrient deficiency, inflammation and malarial antigenemia
Source: PLoS One. 2021 Nov 4;16(11):e0259509. doi: 10.1371/journal.pone.0259509 (PMC8568126; doi:10.1371/journal.pone.0259509)
Supplement: S3 Table — AGP, α-1-acid glycoprotein; CRP, C-reactive protein; HRP2, histidine rich protein 2; N/A, not available; RBP4, retinol binding protein 4; sTfR, soluble transferrin receptor; Tg, thyroglobulin; LK, Liquichek LK; SLK, spiked Liquichek. After each analyte in parentheses are the LLOQ and ULOQ, respectively. (DOCX) [file pone.0259509.s003.docx]

**S3 Table. Assay precision and linearity for 7 plex array.**

| .e | Statistic | LK low | LK med | LK high | SLK low | SLK med | SLK high | Serum 1 | Serum 2 | Serum 3 | Serum 4 | Serum 5 | Serum 6 | Serum 7 |
| --- | --- | --- | --- | --- | --- | --- | --- | --- | --- | --- | --- | --- | --- | --- |
| AGP  (0.001-0.37 g/L) | Mean | 0.012 | 0.029 | 0.092 | 0.012 | 0.028 | 0.103 | 0.073 | 0.074 | 0.088 | 0.153 | 0.137 | 0.084 | 0.08 |
|  | Intra assay CV (%) | 1.9 | 5.03 | 5.41 | 1.72 | 1.21 | 2.4 | 2.49 | 3.65 | 3.23 | 6.2 | 3.28 | 3.58 | 3 |
|  | Inter assay CV (% | 13.73 | 13.85 | 11.44 | 12.18 | 12.34 | 9.49 | 6.52 | 4.34 | 5.39 | 5.54 | 7.06 | 5.7 | 7.45 |
|  | N (plates) | 18 | 20 | 20 | 18 | 20 | 20 | 20 | 20 | 20 | 20 | 20 | 20 | 20 |
|  | Linearity | 98% | | | 99% | | | N/A | N/A | N/A | N/A | N/A | N/A | N/A |
| CRP  (0.028-20.5 mg/L) | Mean | 1.84 | 4.55 | 16.67 | 0.92 | 2.17 | 8.36 | 0.15 | 0.26 | 0.39 | 0.28 | 3.1 | 0.7 | 0.26 |
|  | Intra assay CV (%) | 1.47 | 1.84 | 4.41 | 1.75 | 1.74 | 3.63 | 6.05 | 4.32 | 2.35 | 2.09 | 2.74 | 2.08 | 3.22 |
|  | Inter assay CV (% | 13.13 | 13.85 | 9.21 | 10.87 | 12.79 | 10.45 | 11.35 | 9.73 | 11.42 | 9.12 | 8.3 | 7.62 | 10.92 |
|  | N (plates) | 20 | 20 | 18 | 19 | 20 | 20 | 18 | 18 | 18 | 18 | 20 | 19 | 18 |
|  | Linearity | 98% | | | 99% | | | N/A | N/A | N/A | N/A | N/A | N/A | N/A |
| Ferritin  (0.156-114 μg/L) | Mean | 26.43 | 58.54 | 97.86 | 32.44 | 74.34 | 95.87 | 8.13 | 0.88 | 0.72 | 0.44 | 3.06 | 0.41 |  |
|  | Intra assay CV (%) | 1.53 | 1.69 | 3.77 | 2.45 | 2.62 | 2.19 | 3.02 | 5.81 | 8.66 | 7.99 | 2.76 | 15.66 |  |
|  | Inter assay CV (% | 12.23 | 14.7 | 8.53 | 12.03 | 12.81 | 9.81 | 6.82 | 14.27 | 16.32 | 14.15 | 11.73 | 3.06 |  |
|  | N (plates) | 20 | 20 | 8 | 20 | 17 | 9 | 20 | 18 | 17 | 14 | 20 | 14 | 0 |
|  | Linearity | 83% | | | 57% | | | N/A | N/A | N/A | N/A | N/A | N/A | N/A |
| HRP2  (0.001-1.04 μg/L) | Mean | N/A | N/A | N/A | 0.05 | 0.13 | 0.45 | N/A | N/A | N/A | N/A | N/A | N/A | N/A |
|  | Intra assay CV (%) | N/A | N/A | N/A | 1.46 | 1.9 | 2.58 | N/A | N/A | N/A | N/A | N/A | N/A | N/A |
|  | Inter assay CV (% | N/A | N/A | N/A | 10.23 | 8.81 | 7.24 | N/A | N/A | N/A | N/A | N/A | N/A | N/A |
|  | N (plates) | N/A | N/A | N/A | 20 | 20 | 20 | N/A | N/A | N/A | N/A | N/A | N/A | N/A |
|  | Linearity |  | N/A |  | 99% | | | N/A | N/A | N/A | N/A | N/A | N/A | N/A |
| RBP4  (0.001-1.04 μmol/L) | Mean | 0.032 | 0.077 | 0.326 | 0.019 | 0.047 | 0.181 | 0.224 | 0.277 | 0.181 | 0.226 | 0.262 | 0.274 | 0.227 |
|  | Intra assay CV (%) | 1.19 | 1.53 | 3.35 | 1.98 | 1.49 | 2.84 | 5.11 | 5.37 | 7.47 | 4.34 | 2.57 | 4.33 | 4.9 |
|  | Inter assay CV (% | 12.78 | 13.61 | 14.18 | 11.45 | 11.62 | 11.42 | 8.07 | 7.62 | 6.92 | 7.19 | 7.41 | 9.8 | 9.17 |
|  | N (plates) | 18 | 20 | 20 | 18 | 18 | 20 | 20 | 20 | 20 | 20 | 20 | 20 | 20 |
|  | Linearity | 99% | | | 99% | | | N/A | N/A | N/A | N/A | N/A | N/A | N/A |
| sTfR  (0.163-119 mg/L) | Mean | 0.25 | 0.58 | 1.53 | 1.36 | 2.81 | 7.24 | 1.42 | 1.45 | 1.46 | 1.02 | 2.43 | 1.3 | 1.89 |
|  | Intra assay CV (%) | 6.39 | 6.57 | 4.75 | 2.8 | 1.74 | 2.63 | 4.04 | 5.77 | 3.15 | 4.24 | 2.89 | 3.07 | 3.29 |
|  | Inter assay CV (% | 16.8 | 22.59 | 33.9 | 13.52 | 14.77 | 18.06 | 9.2 | 10.86 | 11.2 | 13.37 | 12.13 | 9.96 | 9.78 |
|  | N (plates) | 14 | 16 | 18 | 18 | 18 | 18 | 18 | 18 | 18 | 18 | 18 | 18 | 18 |
|  | Linearity | 96% | | | 98% | | | N/A | N/A | N/A | N/A | N/A | N/A | N/A |
| Tg  (0.019-13.7 μg/L) | Mean | 0.03 | 0.11 | 0.4 | 0.64 | 1.72 | 8.7 | N/A | N/A | 2.2 | 1.46 | 3.53 | 1.01 | 0.38 |
|  | Intra assay CV (%) | 9.14 | 3.71 | 3.05 | 2.07 | 1.53 | 5.36 | N/A | N/A | 2.41 | 3.93 | 2.37 | 2.97 | 2.3 |
|  | Inter assay CV (% | 23 | 23.74 | 12.92 | 10.75 | 10.99 | 19.28 | N/A | N/A | 14.3 | 11.61 | 15.91 | 11.15 | 14.9 |
|  | N (plates) | 7 | 18 | 18 | 19 | 20 | 20 | 0 | 20 | 20 | 20 | 20 | 20 | 18 |
|  | Linearity | 98% | | | 98% | | | N/A | N/A | N/A | N/A | N/A | N/A | N/A |

AGP, α-1-acid glycoprotein; CRP, C-reactive protein; HRP2, histidine rich protein 2; N/A, not available; RBP4, retinol binding protein 4; sTfR, soluble transferrin receptor; Tg, thyroglobulin; LK, Liquichek LK; SLK, spiked Liquichek. After each analyte in parentheses are the LLOQ and ULOQ respectively.
