# Supplementary material for: A multicenter analytical performance evaluation of a multiplexed immunoarray for the simultaneous measurement of biomarkers of micronutrient deficiency, inflammation and malarial antigenemia
Source: PLoS One. 2021 Nov 4;16(11):e0259509. doi: 10.1371/journal.pone.0259509 (PMC8568126; doi:10.1371/journal.pone.0259509)
Supplement: S4 Table — AGP, α-1-acid glycoprotein; CRP, C-reactive protein; HRP2, histidine rich protein 2; RBP4, retinol binding protein 4; sTfR, soluble transferrin receptor; Tg, thyroglobulin. (DOCX) [file pone.0259509.s004.docx]

**S4 Table.** The upper- and lower limits of quantification for each biomarker using the 7-plex assay.

|  | AGP (g/L) | CRP (mg/L) | Ferritin (µ/L) | HRP2 (µg/L) | RBP4 (µmol/L) | sTfR (mg/L) | Tg (µg/L) |
| --- | --- | --- | --- | --- | --- | --- | --- |
| Upper Limit of Quantification | 4.3 | 91.4 | 1011 | 6.9 | 9.3 | 1210 | 122 |
| Lower Limit of Quantification | 0.0083 | 0.14 | 1.7 | 0.011 | 0.024 | 1.8 | 0.19 |

AGP, α-1-acid glycoprotein; CRP, C-reactive protein; HRP2, histidine rich protein 2; RBP4, retinol binding protein 4; sTfR, soluble transferrin receptor; Tg, thyroglobulin.
