# Supplementary figures and images for: A multicenter analytical performance evaluation of a multiplexed immunoarray for the simultaneous measurement of biomarkers of micronutrient deficiency, inflammation and malarial antigenemia
Source: PLoS One. 2021 Nov 4;16(11):e0259509. doi: 10.1371/journal.pone.0259509 (PMC8568126; doi:10.1371/journal.pone.0259509)

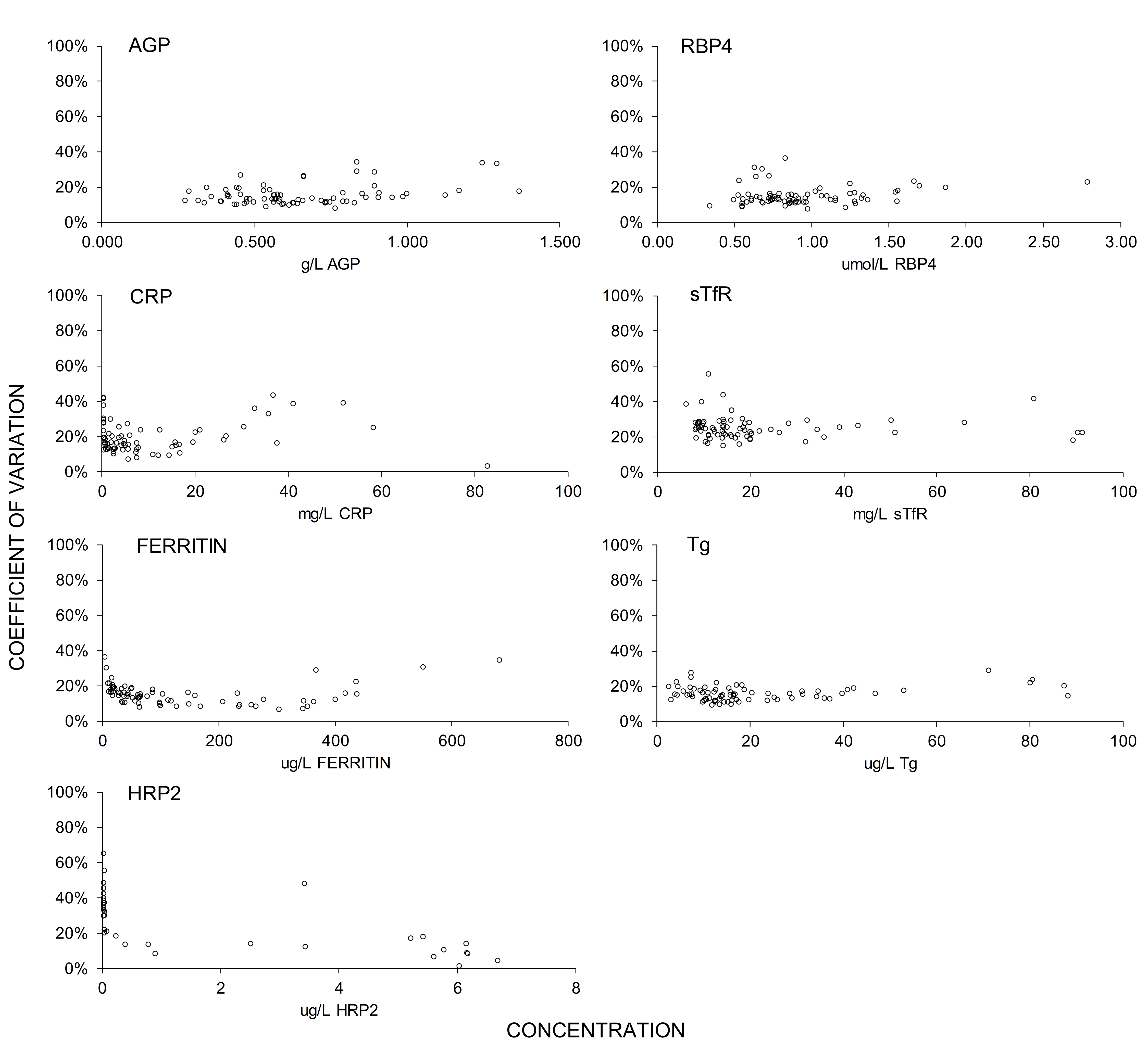

Supplement: S1 Fig — Y-axes set to 100% for all analytes. One CRP result (31 mg/L, CV = 123%) and one HRP2 result (0.2 mg/mL, CV = 177%) are not shown. AGP, α-1-acid glycoprotein; CRP, C-reactive protein; HRP2, histidine rich protein 2; RBP4, retinol binding protein 4; sTfR, soluble transferrin receptor; Tg, thyroglobulin. (TIF) [file pone.0259509.s005.tif]
