# Supplementary material for: A multicenter analytical performance evaluation of a multiplexed immunoarray for the simultaneous measurement of biomarkers of micronutrient deficiency, inflammation and malarial antigenemia
Source: PLoS One. 2021 Nov 4;16(11):e0259509. doi: 10.1371/journal.pone.0259509 (PMC8568126; doi:10.1371/journal.pone.0259509)
Supplement: Attachment — Submitted filename: Review PLOSOne.docx [file pone.0259509.s006.docx]

**Manuscript Number PONE-D-21-06971
"A multicenter analytical performance evaluation of a multiplexed immunoarray for the simultaneous measurement of biomarkers of micronutrient deficiency, inflammation and malarial antigenemia"**

The work by Brindle et al. is quite well written and extensively describes a 7-plex ELISA-based assay for the simultaneous detection of several circulating biomarkers for malaria, inflammation and malnutrition. However, some aspect of the paper could be clarified or improved:

1.	In the abstract, authors say that this immunoassay can be used on both plasma and serum, however, successively authors only describe plasma analysis they performed: did you test it also in serum? If yes, please show results in a supplementary material file in order to demonstrate that both biofluid can be indifferently used or explain why they chose plasma (is it more reproducible? Is there some molecule not detectable or highly confounding in serum measurement?). If serum has not been evaluated, please remove it.

2.	Since followed workflow is quite long, I strongly suggest to authors to describe it in a graphical workflow, in order to allow readers to catch key points of primary analysis and validation.

3.	Patients nor controls have been described from a clinical point of view: are they comparable? Please, provide a table containing at least basic clinical characteristics of analyzed subjects (variability in molecules detection could also derive from heterogeneity of population?).

4. In Materials and Methods section authors start with "validation". However, it is impossible to start with a validation. I suggest to modify this term and substitute it with "Test" or "Setup".

5.	Authors did not explain why a 7-plex only considering plasma evaluation. Could analysis of other molecules (in other biofluids) such as uric Nitrogen (evaluation of protein intake) or erythrocyte EPA (eicopentanoid acid, for omega-3 intake) render this assay more reliable and reproducible?

6.	Which is the clinical relevance of this study? It is very important to demonstrate the reproducibility of this test, however, in order to render more relevant the study from a clinical point of view, I strongly suggest to authors to consider adding, in discussion section, some sentence about the clinical applicability and importance of this test. For example: this test will be potentially used prevalently in countries with a high rate of malnutrition and a high prevalence of malaria, which are not very rich countries: which is the cost of this assay? Is it applicable to villages hard to access? Which is the time for referral? Please, describe advantages and disadvantages.

7.	Please, provide spelling of some abbreviation and revise some English expression.
